# A novel training program: laparoscopic versus robotic-assisted low anterior resection for rectal cancer can be trained simultaneously

**DOI:** 10.3389/fonc.2023.1169932

**Published:** 2023-06-27

**Authors:** Yanlei Wang, Dongpeng Wen, Cheng Zhang, Zhikai Wang, Jiancheng Zhang

**Affiliations:** Department of Gastrointestinal Surgery, Henan Provincial People’s Hospital, Zhengzhou University People’s Hospital, Henan University People’s Hospital, Zhengzhou, China

**Keywords:** robotic, da Vinci, learning curve, laparoscopic, low anterior resection

## Abstract

**Background:**

Current expectations are that surgeons should be technically proficient in minimally invasive low anterior resection (LAR)—both laparoscopic and robotic-assisted surgery. However, methods to effectively train surgeons for both approaches are under-explored. We aimed to compare two different training programs for minimally invasive LAR, focusing on the learning curve and perioperative outcomes of two trainee surgeons.

**Methods:**

We reviewed 272 consecutive patients undergoing laparoscopic or robotic LAR by surgeons A and B, who were novices in conducting minimally invasive colorectal surgery. Surgeon A was trained by first operating on 80 cases by laparoscopy and then 56 cases by robotic-assisted surgery. Surgeon B was trained by simultaneously performing 80 cases by laparoscopy and 56 by robotic-assisted surgery. The cumulative sum (CUSUM) method was used to evaluate the learning curves of operative time and surgical failure.

**Results:**

For laparoscopic surgery, the CUSUM plots showed a longer learning process for surgeon A than surgeon B (47 vs. 32 cases) for operative time, but a similar trend in surgical failure (23 vs. 19 cases). For robotic surgery, the plots of the two surgeons showed similar trends for both operative times (23 vs. 25 cases) and surgical failure (17 vs. 19 cases). Therefore, the learning curves of surgeons A and B were respectively divided into two phases at the 47th and 32nd cases for laparoscopic surgery and at the 23rd and 25th cases for robotic surgery. The clinicopathological outcomes of the two surgeons were similar in each phase of the learning curve for each surgery.

**Conclusions:**

For surgeons with rich experience in open colorectal resections, simultaneous training for laparoscopic and robotic-assisted LAR of rectal cancer is safe, effective, and associated with accelerated learning curves.

## 1 Introduction

Since the laparoscope was first applied in colorectal disease in 1991 (1), laparoscopic colorectal surgery has gained worldwide popularity as a method for the local excision of rectal neoplasms. Numerous studies, including the famous ACOSOG and ALaCaRT trials, have shown that laparoscopic rectal resection can improve short-term outcomes, leading to smaller incisions, lower wound infection rates, less blood loss, and faster recovery, compared to conventional open surgery (2–4). Furthermore, a meta-analysis including 12 randomized clinical trials demonstrated that laparoscopic rectal cancer resection did not compromise oncological outcomes (5). However, the long, nonergonomic surgical instruments with two-dimensional vision in a narrow pelvic cavity make laparoscopic total mesorectal excision a technically demanding procedure, resulting in steep learning curves for surgeons, and has seriously limited its adoption (6). The da Vinci Surgical System™ (Intuitive Surgical^®^, Sunnyvale, CA, USA), as a novel minimally invasive approach, overcomes the disadvantages of the laparoscope. Its technical advantages, including ergonomically designed surgical instruments, can not only translate into clinical benefits for patients, such as lower conversion rate, shorter hospital stay, reduced complications, and faster recovery in urogenital function (7–9). Additionally, they can shorten the learning curve for surgeons (10).

At present, both minimally invasive approaches have been applied in many medical centers. As laparoscopic and robotic surgery are popular among most patients with rectal cancer, surgeons must be technically proficient in both approaches. Presently, the most common training program (“standard program”) involves teaching laparoscopy first, followed by robotics, after achieving proficiency in laparoscopy. However, effective methods for training surgeons to be skilled using both approaches are relatively unexplored. Previous literature reports have shown that prior experience in laparoscopic rectal surgery can facilitate the learning curve for robotic rectal resection (11, 12) and an accelerated rectal resection learning curve can be attained for open and laparoscopic approaches simultaneously (13). Therefore, experience using one approach can facilitate the process of learning for other techniques. Furthermore, a study has shown that prior laparoscopic experience is not always essential for implementing a robotic program (14). Hence, we hypothesized that surgeons can be simultaneously trained to perform the two approaches for rectal resection (referred to as the “novel program”) and rapidly achieve proficiency in both.

This retrospective study aimed to compare two different training programs (“standard” and “novel” programs) for learning minimally invasive low anterior resection (LAR) for rectal cancer, focusing on the learning curves and perioperative outcomes of two trainee surgeons, who were novices in minimally invasive colorectal surgery.

## 2 Patients and methods

### 2.1 Patients and study design

**Figure 1:**
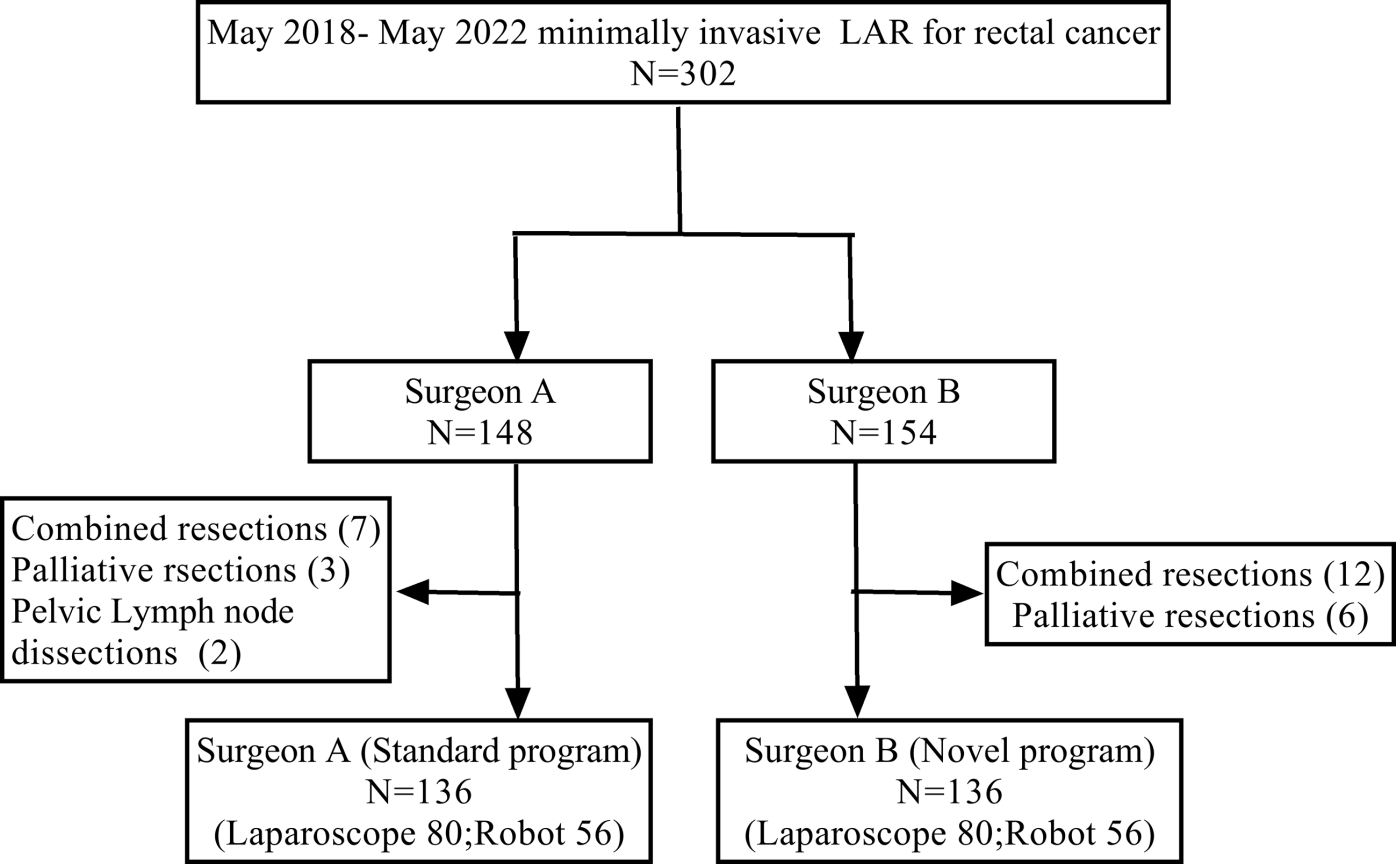
Flow chart of patient selection.

Data from patients with histologically confirmed rectal adenocarcinoma treated at the Henan Provincial People’s Hospital from May 2018 to May 2022 were retrospectively reviewed. Overall, 302 consecutive patients who underwent minimally invasive LAR by surgeons A and B were initially included. After excluding 12 cases operated by surgeon A (7 combined resections, 3 palliative resections, and 2 pelvic lymph node dissections) and 18 cases by surgeon B (12 combined resections and 6 palliative resections), 136 cases were finally enrolled for each surgeon, including 80 laparoscopic and 56 robotic cases, respectively (**Figure 1**). The detailed characteristics of the robotic and the laparoscopic procedures were explained to the patients before surgery and they were informed about patients how much they would have to pay for each operation. The final choice was made by shared decision-making between patients and operating teams. All patients were followed up for at least 6 months.

Surgeons A and B performed their first laparoscopic LAR (L-LAR) in May 2018 and April 2019, and then performed their first robotic LAR (R-LAR) in August 2019 and June 2019, respectively. During the study period, surgeon A performed the first 80 laparoscopic cases and then the 56 robotic cases using the da Vinci surgical robot (“standard program”); while surgeon B performed an initial 15 cases using the laparoscope and then the remaining 121 cases using the laparoscope and the da Vinci surgical robot alternately (“novel program”). The two surgeons had similar case volumes (L-LAR and R-LAR) per year.

Analyzed data included patient demographics, preoperative assessment, intra- and postoperative outcomes, and pathological parameters. Rigid colonoscopy with biopsy and pelvic magnetic resonance imaging (MRI) were performed as routine examinations before the operation. Preoperative chemoradiotherapy (CRT) was recommended for patients with locally advanced rectal cancer (cT3–4 and/or N+) based on MRI examination. Final decisions were made in multidisciplinary treatment meetings. Operative time (OT) was from skin incision to closure. Surgeon console time was the time the surgeon used the robot. Docking time was the time to position the robot and secure the arms to the ports. Postoperative complications included only anastomosis-related complications, such as anastomotic leakage or bleeding. Complications ≥ grade III, according to the Clavien–Dindo classification, were considered severe. Circumferential resection margin (CRM) and distal resection margin (DRM) were considered involved if malignant cells were found within ≤ 1 mm from the CRM or DRM. Surgical failure was defined as the presence of one or more of the following four parameters: conversion, R1 resection (CRM or DRM involved), severe anastomosis-related complications (≥ Clavien–Dindo grade III), and number of harvested lymph nodes< 12. Otherwise, the case was considered a surgical success.

This study was approved by the Ethical Committee of the Henan Provincial People’s Hospital and written informed consent was obtained from all patients.

### 2.2 Surgical procedure

For R-LAR, all cases were performed using the da Vinci Si surgical system. A totally robotic technique was adopted in all cases, and standardized as follows. After pneumoperitoneum was established, the assistant docked the robot. The inferior mesenteric vessels were first ligatured and then, the left colon and sigmoid colon were dissected in a medial-to-lateral approach. The splenic flexure was taken down, if appropriate. Then, the pelvic cavity was dissected along the nonvascular plane between the parietal fascia and the visceral fascia of the pelvis, first by a posterior dissection, followed by lateral and anterior dissections. The robot was undocked when the dissection was completed. The rectum was transected laparoscopically and an intracorporeal end-to-end anastomosis was constructed. The specimen was extracted through a Pfannenstiel incision using a wound protector. An ileostomy was created during the operation on the decision of the surgeon when the following criteria were met: uncertainty of the blood supply of the anastomosed stoma, tension in the anastomosed stoma, and poor nutritional status. L-LAR was performed in the same manner as described above for R-LAR.

### 2.3 Surgeon training and standardization

Both surgeons had approximately 10 years of experience in the Department of Gastrointestinal Surgery. During this period, they had undergone training for open colorectal resections, under the supervision of an experienced surgeon and performed open colorectal resections independently on > 200 cases. Before performing the first L-LAR, they received rigorous dry laboratory training in laparoscopy and then participated in colorectal resections as assistants during fellowship training, with surgeons A and B assisting in 50 and 58 cases, respectively. Then, they performed laparoscopic operations for simple surgeries, followed by more difficult surgeries, such as LAR. Surgeons were similarly trained in the robotic procedure during the fellowship training period: first, dry laboratory training; then, assisting robotic colorectal surgeries (47 and 42 cases for surgeons A and B, respectively); and finally, using the robot independently. The initial cases conducted by the two surgeons were supervised by the previous surgeon who had rich experience in laparoscopic and robotic colorectal resections. He monitored and advised on surgical tips for the manipulation of the instruments and technical tips for laparoscopic and robotic surgeries. After approximately 20 cases, surgeons A and B could perform the operations independently, without supervision.

### 2.4 CUSUM method

Learning curves were analyzed using the cumulative sum (CUSUM) method, based on two parameters: OT and surgical failure, with the following equation:


CUSUM= ∑(Xi–Xo)

where *Xi* is an individual value, and *X_0_* is the mean of the overall value for each surgeon.

For example, concerning OT, all cases were arranged in chronological order for each surgeon. The CUSUM_OT_ for the first case was the difference between the OT for the first case and the mean OT of all cases for each surgeon. The CUSUM_OT_ of the second case was the CUSUM_OT_ of the previous case added to the difference between the OT of the second case and the mean OT for all cases for each surgeon. This procedure was repeated for each patient except the last one, which was set as zero. For surgical failure, *Xi* was an individual attempt and *X_0_* was the observed failure rate of each approach for each surgeon, with *Xi* assigned a score of 0 for success and 1 for failure.

CUSUM curves ascended when the set value was not reached, which reflected an ongoing learning process; otherwise, the performance was more often on target than expected. Curve analysis involved the identification of the turning point at which the graph adopted a general downward slope; this point represents the end of the initial learning process, after which the target value began to be observed.

### 2.5 Statistical analysis

All statistical analyses were conducted using SPSS statistical software (version 22; IBM Corporation, Armonk, NY, USA). Categorical variables were compared using chi-squared or Fisher’s exact tests. Continuous variables are presented as mean ± standard deviation or median (range) and were compared using an independent two-sample *t* test or the Mann–Whitney *U* test, as appropriate. *P<* 0.05 was considered statistically significant.

## 3 Results

### 3.1 Patient characteristics

**Figure 2:**
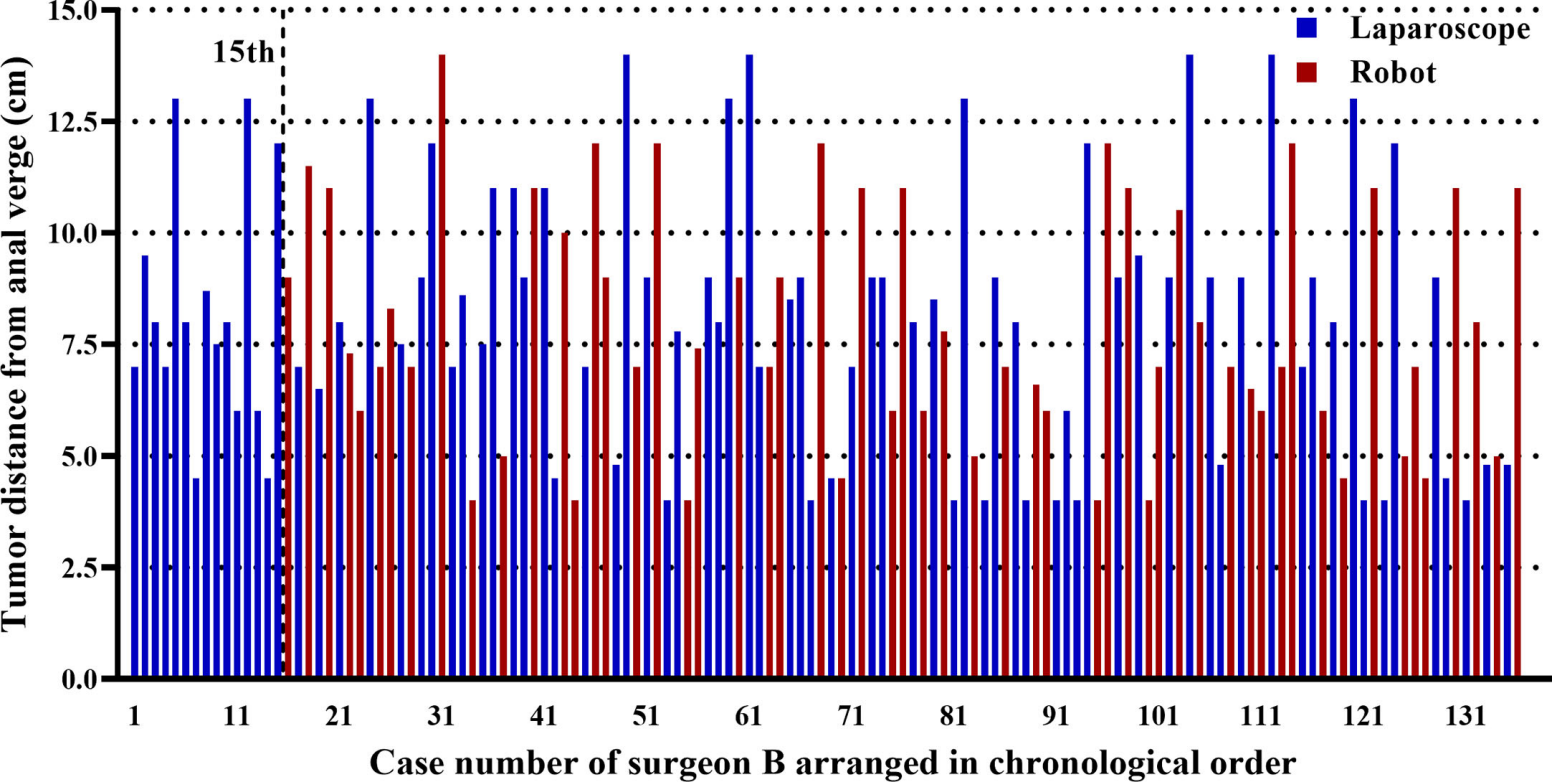
Graph representing the tumor location in each of the cases of surgeon B arranged in chronological order. The first 15 laparoscopic cases were operated before the first robotic case, and the remaining 121 cases (65 laparoscopic and 56 robotic cases) were operated using both approaches alternately.

Patients were matched for each approach according to age, sex, body mass index (BMI), American Society of Anesthesiologists score (ASA), tumor location, and CRT (*p* > 0.05) (**Table 1**). In addition, the mean tumor location from the anal verge was 8.0 cm for all the 136 cases of surgeon B. **Figure 2** demonstrates this parameter for each case in chronological order.

**Table 1:** Patient characteristics and clinicopathologic outcomes.

	Laparoscopy (n=160)			Robot (n=112)			
	Surgeon A (n=80)	Surgeon B (n=80)	*p*	Surgeon A (n=56)	Surgeon B (n=56)		*p*
Age (year)	60.8±12.5	58.6±13.0	0.292	60.3±10.9	57.9±11.8		0.262
Sex (male)	40 (50.0)	38 (47.5)	0.752	37 (66.1)	34 (60.7)		0.556
BMI (kg/m^2^)	23.0±4.3	22.5±3.7	0.449	23.6±3.1	22.7±2.7		0.146
ASA score, I/II/III	26/34/20(32.5/42.5/25.0)	27/26/27(33.8/32.5/33.8)	0.345	10/25/21(17.9/44.6/37.5)	15/23/18(26.8/41.1/32.1)		0.518
Distance from AV (cm)	7.5±2.9	8.2±3.0	0.135	7.3±2.6	7.9±2.7		0.239
CRT	32 (40.0)	25 (31.3)	0.248	23 (41.1)	26 (46.4)		0.568
Operative time (min)	305.3±45.5	293.3±42.3	0.085	324.1±51.5	333.8±50.0		0.317
Surgeon console time (min)	–	–	–	249.2±48.3	258.8±46.9		0.240
Docking time (min)	–	–	–	6.9±3.1	7.1±2.8		0.658
Blood loss (ml)	177.4±72.3	187.8±83.4	0.402	142.9±86.6	129.8±56.0		0.346
Conversion	2 (2.5)	3 (3.8)	1.000	2 (3.6)	1 (1.8)		1.000
Ileostomy	46 (57.5)	35 (43.8)	0.114	32 (57.1)	27 (48.2)		0.344
Hospital stay (day)	11.2±2.7	12.5±4.2	0.019	10.7±4.5	9.4±3.4		0.162
Clavien-Dindo III–IV	3 (3.8)	2 (2.5)	1.000	2 (3.6)	3 (5.4)		1.000
pTNM stage, I/II/III	22/20/38(27.5/25.0/47.5)	19/21/40(23.8/26.3/50.0)	0.863	13/19/24(23.2/33.9/42.9)	15/17/24(26.8/30.4/42.9)		0.881
Retrieved LN	16.0±4.6	17.7±5.2	0.029	14.7±3.8	16.0±4.3		0.102
Retrieved LN <12	10 (12.5)	7 (8.8)	0.442	5 (8.9)	5 (8.9)		1.000
Tumor size (cm)	3.9±2.0	3.6±1.5	0.325	3.3±1.2	3.7±1.5		0.208
DRM (cm)	2.6±1.5	3.0±1.8	0.094	1.8±1.3	2.2±1.3		0.140
DRM involved	0	0	–	1 (1.8)	0		1.000
CRM involved (<1.0mm)	1 (1.3)	2 (2.5)	1.000	0	2 (3.6)		0.495
Surgical failure	13 (16.3)	11 (13.8)	0.658	7 (12.5)	8 (14.3)		0.781	

Values are presented as mean ± standard deviation, or n (%). BMI, body mass index; ASA, American Society Anesthesia; AV, anal verge; CRT, chemoradiation therapy; LN, lymph node; DRM, distal resection margin; CRM, circumferential resection margin.

### 3.2 Clinicopathologic outcomes

For laparoscopic surgery, OT values were similar between surgeons A and B (305.3 vs. 293.3 min, *p* = 0.085) (**Table 1**). Blood loss for surgeon A was 10 mL less than that for surgeon B; however, this difference was not statistically significant (177.4 vs. 187.8 mL, *p* = 0.402). Two conversions occurred for surgeon A and three for surgeon B, due to insufficient experience (2.5% vs. 3.8%, *p* = 1.000). There were three severe anastomosis-related complications (two anastomotic leakages and one anastomotic bleeding) for surgeon A and two (one anastomotic leakage and one anastomotic bleeding) for surgeon B (*p* = 1.000), all of which were treated by endoscopy. Patients operated by surgeon A had significantly shorter hospital stays (11.2 vs. 12.5 days, *p* = 0.019) and fewer lymph nodes (16.0 vs. 17.7, *p* = 0.029) than those operated by surgeon B. All three patients with positive CRM received postoperative chemoradiotherapy and no local recurrence was observed during follow-up. Surgical failure rates were comparable between the two surgeons (16.3% vs. 13.8%, *p* = 0.658).

For the robotic approach, there were no significant differences between the two surgeons in any parameters (*p* > 0.05) (**Table 1**).

### 3.3 Learning curve analysis

**Figure 3:**
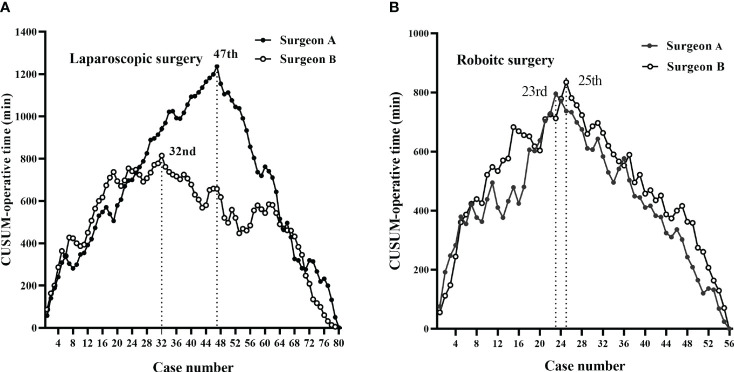
CUSUM plots for total operative time. The learning curves ascended during the initial phase, followed by a peak and then a gradual descent. **(A)** The laparoscopic surgery learning curve of surgeon A (47 cases) was longer than that of surgeon B (32 cases). **(B)** The robotic-assisted surgery learning curves of the two surgeons were similar (23 vs. 25 cases for surgeon A vs. surgeon B).

**Figure 4:**
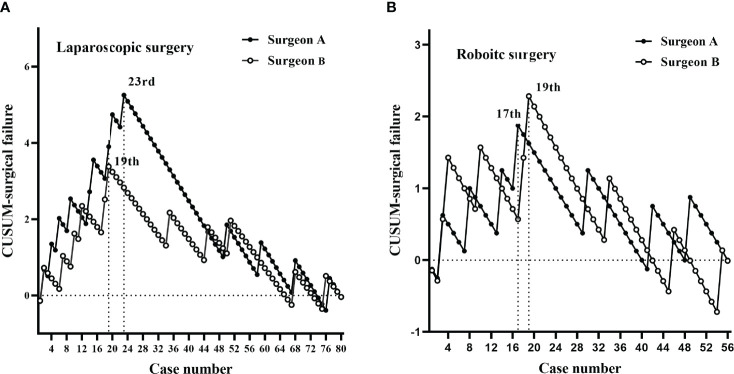
CUSUM plots for surgical failure. The plots for each surgery showed similar trends for both surgeons. **(A)** Laparoscopic surgery peaks for surgeons A and B were at the 23^rd^ and 19^th^ cases, respectively. **(B)** Robotic-assisted surgery peaks for surgeons A and B were at the 17^th^ and 19^th^ cases, respectively.

According to CUSUM analysis, the learning curves ascended at the initial phase, followed by a peak, and then a gradual descent (**Figures 3**, **4**). For laparoscopic surgery, the OT learning curve was longer for surgeon A (47 cases) than for surgeon B (32 cases). Regarding surgical failure, peak points were at the 23^rd^ and 19^th^ cases, respectively, indicating a shorter learning process than that of OT. Therefore, the CUSUM_OT_ was taken as a surrogate to determine the learning curve. Learning curves for surgeon A were determined for phase 1 (the initial learning period, 1^st^–47^th^ cases) and phase 2 (the post-learning period, 48^th^–80^th^ cases); for surgeon B phase 1 (the initial learning period, 1^st^-32^nd^ cases) and phase 2 (the post-learning period, 33^th^-80^th^ cases) (**Figures 3A**, **4A**).

For robotic surgery, the learning curves showed similar trends between the two surgeons, in terms of OT and surgical failure. Accordingly, learning curves were divided into two phases: phase 1 (1^st^–23^rd^ cases) and phase 2 (24^th^–56^th^ cases) for surgeon A; phase 1 (1^st^–25^th^ cases) and phase 2 (26^th^–56^th^ cases) for surgeon B (**Figures 3B**, **4B**).

### 3.4 Comparison of clinicopathological outcomes of laparoscopic surgery between the two surgeons

**Figure 5:**
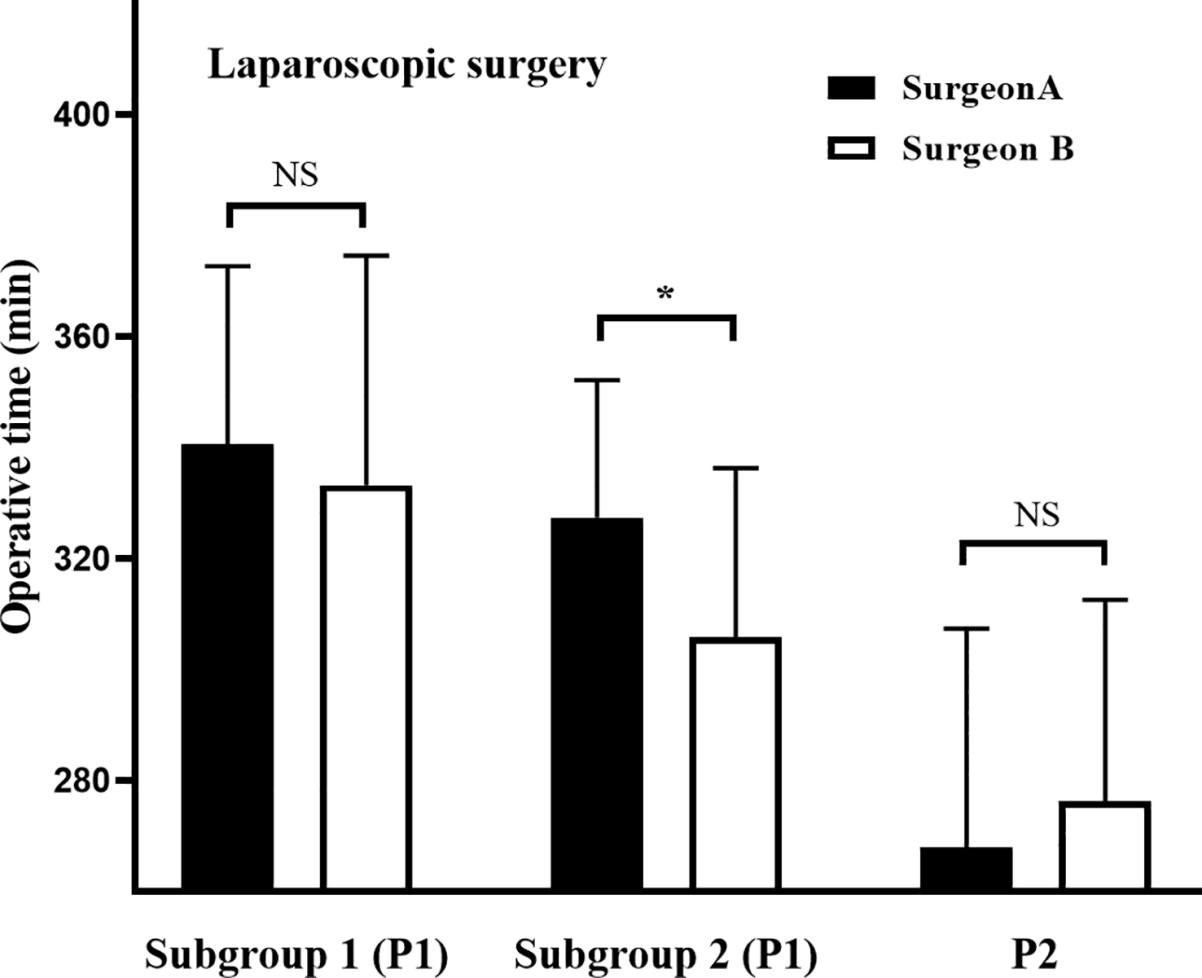
Operative times during different phases of laparoscopic learning curves. The laparoscopic learning curves for phase 1 were divided into subgroup 1 (the first 15 cases) and subgroup 2 (the remaining cases) at case 15 for both surgeons. *P< 0.05 between groups. NS: No Significance.

To fully evaluate the impact of different training programs on clinicopathological outcomes, data were compared according to learning curves (**Table 2**). For phase 1, the OT was 13 min longer for surgeon A than for surgeon B; however, this difference was not statistically significant (331.6 vs. 318.8 min, *p* = 0.106). Considering that surgeon B performed 15 laparoscopic cases before undertaking the first robotic cases, we further divided phase 1 into subgroup 1 (the first 15 cases) and subgroup 2 (the remaining cases) at the 15^th^ case. The results showed that OT was significantly longer for surgeon A than for surgeon B in subgroup 2 (327.4 vs. 305.9 min, *p* = 0.010), while the OT showed no significant difference between surgeons in subgroup 1 (340.7 vs. 333.3 min, *p* = 0.591), phase 2 (267.9 vs. 276.3 min, *p* = 0.325) (**Figure 5**). Other parameters were comparable between the two surgeons (*p* > 0.05) (**Table 2**).

**Table 2:** Clinicopathologic outcomes in laparoscopic surgery between two surgeons.

	Phase 1 (n=79)			Phase 2 (n=81)		
	Surgeon A (n=47)	Surgeon B (n=32)	*p*	Surgeon A (n=33)	Surgeon B (n=48)	*p*
Operative time (min)	331.6±27.6	318.8±38.0	0.106	267.9±39.5	276.3±36.3	0.325
Blood loss (ml)	183.4±58.3	197.5±74.0	0.348	168.8±88.8	181.3±89.2	0.538
Conversion	1 (2.1)	2 (6.3)	0.563	1 (3.0)	1 (2.1)	1.000
Ileostomy	30 (63.8)	15 (46.9)	0.135	16 (48.5)	20 (41.7)	0.544
Hospital stay (day)	11.1±2.5	12.3±3.6	0.098	11.4±3.0	12.7±4.5	0.143
Clavien-Dindo III–IV	2 (4.3)	2 (6.3)	1.000	1 (3.0)	0	0.407
pTNM stage, I/II/III	11/12/24(23.4/25.5/51.1)	5/7/20(15.6/21.9/62.5)	0.571	11/8/14(33.3/24.2/42.4)	14/14/20(29.2/29.2/41.7)	0.920
Retrieved LN	15.7±4.7	17.2±6.2	0.216	16.4±4.5	18.0±4.4	0.120
Retrieved LN <12	6 (12.8)	4 (12.5)	1.000	3 (9.1)	3 (6.3)	0.631
Tumor size (cm)	4.3±2.0	3.6±1.4	0.095	3.4±1.8	3.7±1.6	0.459
DRM (cm)	2.7±1.6	3.4±1.8	0.086	2.4±1.5	2.8±1.7	0.311
DRM involved	0	0	–	0	0	–
CRM involved (<1.0mm)	1 (2.1)	0	1.000	0	2 (4.2)	0.511
Surgical failure	9 (19.1)	6 (18.8)	1.000	4 (12.1)	5 (10.4)	1.000

Values are presented as mean ± standard deviation, or n (%). LN, lymph node; DRM, distal resection margin; CRM, circumferential resection margin.

For phase 2, no parameters differed significantly between the two surgeons (*p*> 0.05) (**Table 2**).

### 3.5 Comparison of clinicopathologic outcomes of robotic surgery between the two surgeons

For phase 1, no significant differences were observed between the two surgeons in terms of OT, surgeon console time, or docking time. The surgical failure rate was 17.4% for surgeon A and 20.0% for surgeon B (*p* = 1.000). For phase 2, 23 patients operated by surgeon A underwent protective ileostomy, compared with 14 operated by surgeon B (69.7% vs 45.2%, *p* = 0.047). Other outcomes were comparable between the two surgeons (*p* > 0.05) (**Table 3**).

**Table 3:** Clinicopathologic outcomes in robotic surgery between two surgeons.

	Phase 1 (n=48)			Phase 2 (n=64)		
	Surgeon A (n=23)	Surgeon B (n=25)	*p*	Surgeon A (n=33)	Surgeon B (n=31)	*p*
Operative time (min)	358.7±55.3	367.2±45.0	0.561	300.0±31.6	306.8±35.6	0.423
Surgeon console time (min)	280.2±49.9	290.4±44.0	0.457	227.6±33.6	235.2±32.6	0.364
Docking time (min)	8.0±4.0	7.4±2.7	0.550	6.1±2.0	6.9±2.9	0.196
Blood loss (ml)	162.6±58.4	141.6±64.0	0.242	129.1±100.3	120.3±47.6	0.660
Conversion	1 (4.3)	1 (4.0)	1.000	1 (3.0)	0	1.000
Ileostomy	9 (39.1)	13 (52.0)	0.371	23 (69.7)	14 (45.2)	0.047
Hospital stay (day)	10.9±4.7	9.1±4.1	0.162	10.6±4.5	9.7±2.9	0.351
Clavien-Dindo III–IV	1 (3.0)	2 (3.2)	1.000	1 (3.0)	1 (3.2)	1.000
pTNM stage, I/II/III	6/5/12(26.1/21.7/52.2)	6/6/13(24.0/24.0/52.0)	0.744	7/14/12(21.2/42.4/36.4)	9/11/11(29.0/35.5/35.5)	0.744
Retrieved LN	14.2±3.2	15.6±4.2	0.199	15.0±4.2	16.2±4.4	0.272
Retrieved LN <12	3 (13.0)	3 (12.0)	1.000	2 (6.1)	2 (6.5)	1.000
Tumor size (cm)	3.2±0.9	3.6±1.3	0.297	3.4±1.4	3.8±1.6	0.393
DRM (cm)	2.2±1.4	2.5±1.4	0.428	1.6±1.2	1.9±1.1	0.228
DRM involved	0	0	–	1 (3.0)	0	1.000
CRM involved (<1.0mm)	0	1 (4.0)	1.000	0	1 (3.2)	0.484
Surgical failure	4 (17.4)	5 (20.0)	1.000	3 (9.1)	3 (9.7)	1.000

Values are presented as mean ± standard deviation, or n (%). LN, lymph node; DRM, distal resection margin; CRM, circumferential resection margin

## 4 Discussion

In most cases, surgeons are trained in laparoscopic and robotic surgery sequentially. However, preliminary experience with laparoscopic LAR is not a necessary pre-requisite for robotic LAR (14). A few studies have reported training for both approaches simultaneously (“novel program”) at the beginning of a surgeon’s minimally invasive career (14–16). Regrettably, these three previous reports on the topic focused on comparing laparoscopic versus robotic surgery by a single surgeon, rather than the training program itself. To provide information on the safety and efficacy of this novel program, we compared it with the “standard program”, in terms of learning curves and perioperative outcomes. The result showed that the novel training program was safe and effective and shortened the learning curve, while not increasing the learning load.

Most patients in this study had mid-high tumors, and a few had low tumors. This was mainly because the tumor distance from the anal verge reflected the depth of dissection in the pelvis and surgical difficulty (17). Low tumors led to increase surgical difficulty and learning load. It was worth pointing out that patients with ultra-low tumor were excluded from our study because they always need transanal total mesorectal excision (TaTME) or partial/total intersphincteric resection (ISR) (18–20). These procedures need additional perineal manipulation, resulting in heterogeneous cases.

The learning curve represents the process of gaining adaptation or becoming skilled in performing a surgical procedure (21), and is influenced by diverse factors, including surgeon characteristics, analytical method, and type of procedure (22). Therefore, to control the bias, the current study included two surgeons with similar surgical experience, who received uniform training and adopted a standardized surgical technique. Furthermore, homogenous cases with rectal cancer undergoing LAR were included in our study. Our data from the two training programs (standard vs novel) showed that the learning curve for robotic surgery (25 vs. 23 cases) was shorter than that for laparoscopic surgery (47 vs. 32 cases). Most previous studies have suggested that the learning curve for rectal cancer surgery was overcome after 15–50 cases for robotic surgery (12, 22–25), and 40–80 cases for laparoscopic surgery (15, 26–28). Hence, there is a consensus that the robotic learning curve is shorter than that of laparoscopy. However, a true comparison of the two learning curves would require surgeons with equal levels of experience with each platform, whereas previous studies involved robotic cases after surgeons had gained extensive laparoscopic experience, making it difficult to draw a conclusion. That is because prior laparoscopic experience can minimize the learning curve for robotic rectal resection (11, 12). Although our team conducted their first robotic case after only 15 cases of laparoscopic experience, the entire learning process was accompanied by laparoscopic cases.

The most important finding was that training on both approaches simultaneously could produce a crossover effect, which can facilitate the learning curves. This study showed that the laparoscopic learning curve was shorter in the simultaneous training program (32 cases) than that in the sequential training program (47 cases), suggesting that the experience gained in robotic surgery can facilitate the laparoscopic learning curve for rectal cancer surgery. Furthermore, considering that the initial 15 laparoscopic cases conducted by surgeon B were trained differently from the other 65 cases, phase 1 was divided into subgroups 1 and 2 at the 15^th^ case. As shown in **Figure 5**, the operative time of the two surgeons was initially similar in subgroup 1, and finally decreased to comparable levels in phase 2, while that of surgeon B decreased faster than that of surgeon A in subgroup 2. Clearly, in subgroup 2, surgeon A was trained for laparoscopic cases and surgeon B for laparoscopic and robotic cases simultaneously. This indicates that training on both approaches simultaneously can rapidly decrease the operative time and accelerate the learning curve, and crossover effects may play a crucial role in these differences. Interestingly, different training programs produced similar robotic learning curves. Indeed, the robotic learning curve of surgeon A was facilitated by his previous laparoscopic experience. Furthermore, the robotic learning curve of surgeon B was also accelerated by the synchronous laparoscopic cases. Therefore, we conclude that the skills attained using both approaches can be mutually translated; that is, there is a crossover effect. This conclusion may be attributable to the similar environment involved in the two approaches, as follows. (i) Both approaches provide a tunnel view, despite the three-dimensional surgical view in robotic surgery. This unfamiliar surgical view is challenging for surgeons, who need more complete views, as provided in conventional open surgery, to establish spatial orientation, which is crucial to minimally invasive surgery (29, 30). (ii) Due to the absence of tactile feedback, visual feedback —such as the pressure exerted on the tissues — plays a significant role in the perception of the intraoperative environment in both approaches (31). (iii) The two surgical approaches comply with the same surgical principles, such as in the identification of anatomical structures and planes of dissection. The sharing of these features between the two approaches produces a crossover effect on the learning curve, where experience gained with either is transferrable to the other, to some extent. However, in our experience, the robot had an obvious advantage over the laparoscope when sewing the anastomotic stoma. During the surgery, the anastomotic wall was shaped by full thickness single-layer continuous suture (32). Unlike the long rigid laparoscopic instruments, the robot was equipped with wristed instrumentation with seven degrees of freedom (DOFs). Therefore, the robot makes sewing more flexible, especially in the narrow and small pelvic cavity.

The poor surgical skills of a surgeon during the initial learning period may not compromise surgical quality. Previous reports have suggested that prolonged operative time for colorectal surgery is associated with worse surgical quality (33, 34). In this study, surgical quality was evaluated using the composite surrogate marker, “surgical failure”, for which the learning curves were shorter than those for the operative time; that is, during the learning process, surgeons first achieved competency for surgical quality, followed by reduced operative time (13). The surgeons had mastered the key points influencing surgical quality in their previous experience of open surgery, helping them to efficiently establish an accelerated surgical failure learning curve for minimally invasive surgery. In our study, the rate of surgical failure ranged from 12.5% to 16.3%. It demonstrated a short learning curve and the rates in phase 2 therefore decreased to 9.1% and 12.1%, which were comparable to the target value of 10% in the literature (35). Therefore, the novel training program can serve as a safe alternative for surgeons. Of the 39 cases of surgical failure, 12 had large tumors located in the mid-low rectum. This relatively low tumor location increased surgical difficulty (17). Likewise, a large tumor in a narrower pelvis is more challenging to expose and dissect, increasing the surgical difficulty. Postoperative CRT was administered for patients with involved surgical margins.

This study had several limitations. First, the results are limited by its retrospective and non-randomized study design. When applying a new technique with which the surgeon has minimal experience, patients with favorable clinical profiles tend to be selected, leading to a bias in the learning curve and clinical outcome data. A randomized controlled trial is necessary to provide deep insight into this novel training program. Second, the first 15 laparoscopic cases conducted by surgeon B were performed before the first robotic case, impairing the consistency of the novel training system. Another limitation was that, during the learning curve, 30 cases of LAR combined with other operations were excluded from this study. These cases could have biased the learning curve to some extent. In addition, only two surgeons were included in this pilot study, which may result in a high risk of selection bias. Therefore, enrolling more surgeons in a future study will provide more robust and convincing data. Despite these limitations, this study provides preliminary insights into the surgical outcomes and learning curves associated with this novel training program.

## 5 Conclusions

For surgeons with rich experience in open colorectal resections, simultaneous training to conduct laparoscopic and robotic-assisted LAR for rectal cancer is safe and effective. This novel training program does not increase the learning load, but does shorten learning curves, which can be attributed to a crossover effect; that is, the skills required for the two approaches are mutually transferrable. Furthermore, surgical outcomes are not compromised. Our results highlight a potential alternative approach for surgeons to rapidly master these two techniques. A randomized controlled trial with more surgeons will provide a more solid methodology for investigating the potential benefits of this novel training program.

## Data availability statement

The raw data supporting the conclusions of this article will be made available by the authors, without undue reservation.

## Ethics statement

The studies involving human participants were reviewed and approved by the Henan Provincial People’s Hospital. The patients/participants provided their written informed consent to participate in this study.

## Author contributions

JZ, ZW, YW, DW, and CZ performed the experiment conception and design. JZ, ZW, YW, DW, and CZ performed the research. YW, DW, and CZ retrieved the data and performed the data collection. YW and DW analyzed the data and did the paper writing. All authors contributed to the article and approved the submitted version.
